# Supplementary material for: Comparative Reverse Vaccinology of Piscirickettsia salmonis, Aeromonas salmonicida, Yersinia ruckeri, Vibrio anguillarum and Moritella viscosa, Frequent Pathogens of Atlantic Salmon and Lumpfish Aquaculture
Source: Vaccines (Basel). 2022 Mar 18;10(3):473. doi: 10.3390/vaccines10030473 (PMC8954842; doi:10.3390/vaccines10030473)
Supplement: Supplementary file 1 [file vaccines-10-00473-s001.zip › Supplementary file S1_MHC II Alpha Chain _ Structure Assessment.pdf]

Structure Assessment

Help Examples

MHC II Alpha Chain; Model 01;

Project Data

Created: Fri 4th Feb, 02:53;

Ramachandran Plots

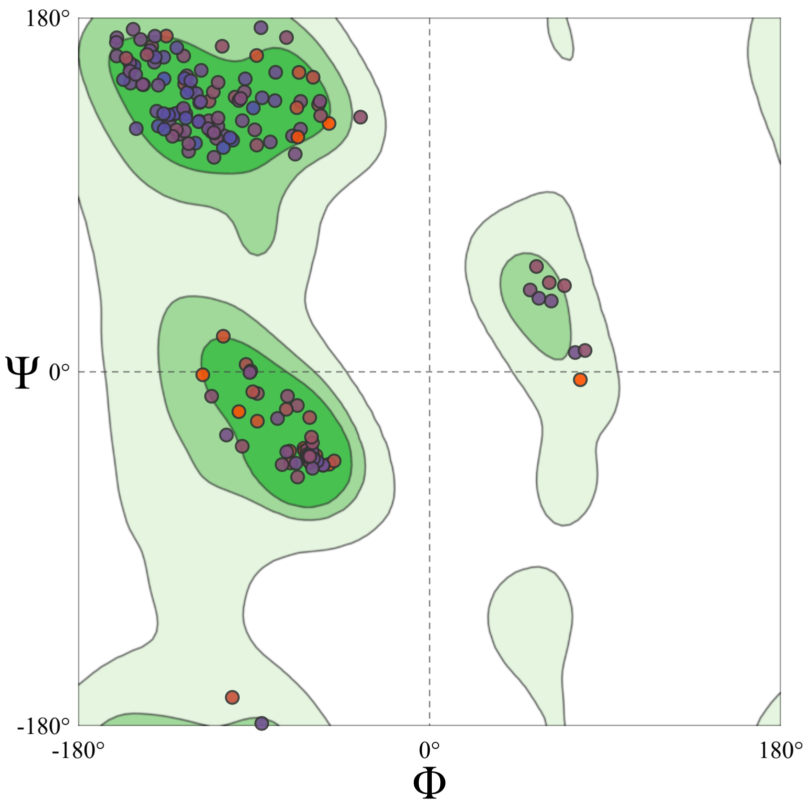

General | Glycine | Proline | Pre-Proline | Chain A

MolProbity Results

MolProbity Score	1.42	
Clash Score	3.71	(A66 TYR-A75 ASP)
Ramachandran Favoured	96.17%	
Ramachandran Outliers	0.00%	

Rotamer Outliers	0.00%	
C-Beta Deviations	3	A199 ALA, A173 GLN, A108 ASN
Bad Bonds	0 / 1565	
Bad Angles	16 / 2136	A80 PHE, (A41 GLY-A42 THR), A110 PRO, A84 ASN, (A109 ASN-A110 PRO), A175 PHE, A29 HIS, A57 VAL, A197 HIS, (A37 CYS-A96 CYS), A94 ASN, A174 THR, (A184 THR-A185 PRO), (A143 TYR-A144 PRO), A75 ASP, A107 GLU
Cis Non-Proline	1 / 170	(A41 GLY-A42 THR)
Cis Prolines	1 / 14	(A143 TYR-A144 PRO)

Results obtained using MolProbity version 4.4

Quality Estimate

QMEANDisCo Global: 0.68 ± 0.06

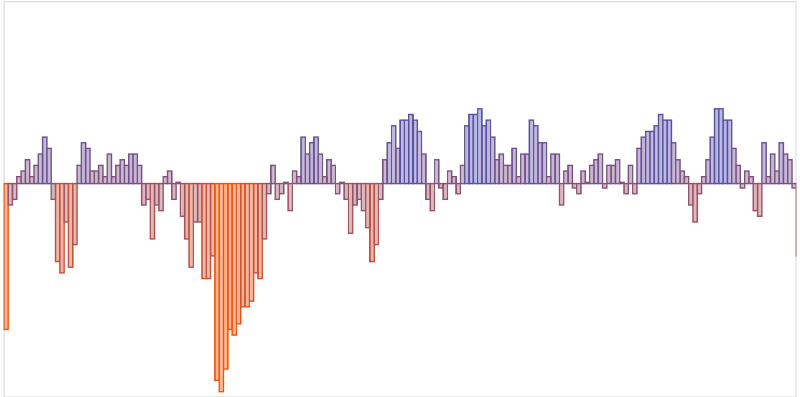

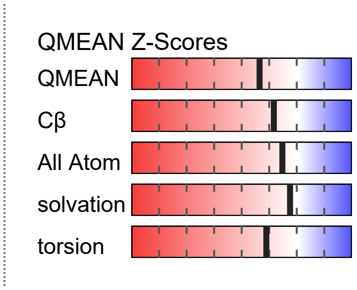

QMEAN	-1.35
Cβ	-0.83
All Atom	-0.52
solvation	-0.24
torsion	-1.10

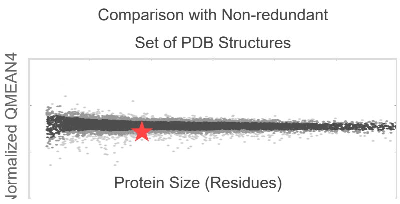

Residue Quality

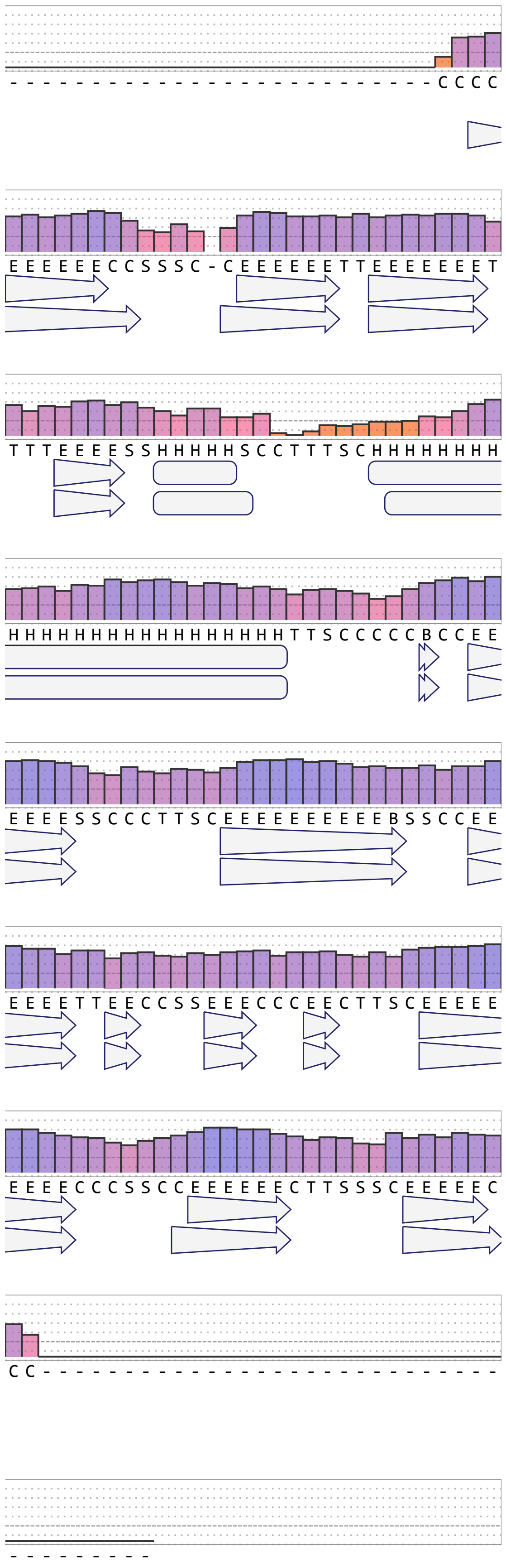

```
    QMEAN                                    30

  Chain:A MKTCSSDMRRSALIIILMINSFCAFSQIPHE
 3wex.1.A --------------------------ADHV     6

    QMEAN

  Chain:A VVYVVGCFENGT-TEAVFEFDAEELLYVDF    59
 3wex.1.A STYAMFVQTHRPTGEFMFEFDEDEQFYVDL    36

    QMEAN

  Chain:A DRDEVVYTMPRFLTDDPSKLFENLNVFNNA    89
 3wex.1.A DKKETVWHLEEFGRAF---SFEAQ----GG    59

    QMEAN

  Chain:A KKNRNICLSIIKIYKLEENNPPEEMDPPES   119
 3wex.1.A LANIAILNNNLNTLIQRSNHTQAANDPPEV    89

    QMEAN

  Chain:A ILYPSEEVQLGVENSLICFVNHFYPPEIKV   149
 3wex.1.A TVFPKEPVELGQPNTLICHIDRFFPPVLNV   119

    QMEAN

  Chain:A SWTRNGRLVSEGQSLSRYYPNNDQTFHQFT   179
 3wex.1.A TWLCNGEPVTEGVAESLFLPRTDYSFHKFH   149

    QMEAN

  Chain:A TLTFTPKEGDIYSCTVEHLALDRPKTRSWV   209
 3wex.1.A YLTFVPSAEDVYDCRVEHWGLDQPLLKHWE   179

    QMEAN

  Chain:A PDFSHPSLGPGVYCGVGLTVGLLGVAVGTF   239
 3wex.1.A AQ----------------------------   181

    QMEAN

  Chain:A LMVKGCRGQ                        248
 3wex.1.A ---------
```

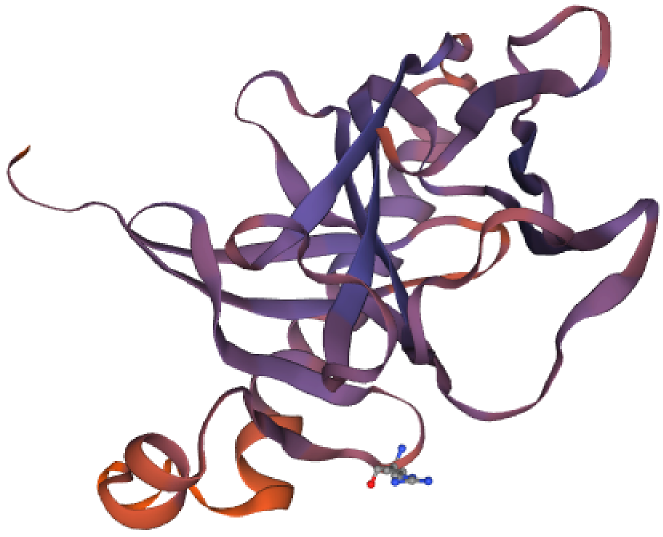

Cartoon
