# Supplementary material for: Comparative Reverse Vaccinology of Piscirickettsia salmonis, Aeromonas salmonicida, Yersinia ruckeri, Vibrio anguillarum and Moritella viscosa, Frequent Pathogens of Atlantic Salmon and Lumpfish Aquaculture
Source: Vaccines (Basel). 2022 Mar 18;10(3):473. doi: 10.3390/vaccines10030473 (PMC8954842; doi:10.3390/vaccines10030473)
Supplement: Supplementary file 1 [file vaccines-10-00473-s001.zip › Supplementary file S2_MHC II Beta Chain _ Structure Assessment.pdf]

Structure Assessment

Help Examples

MHC II Beta Chain; Model 01;

Project Data

Created: Fri 4th Feb, 02:57;

Ramachandran Plots

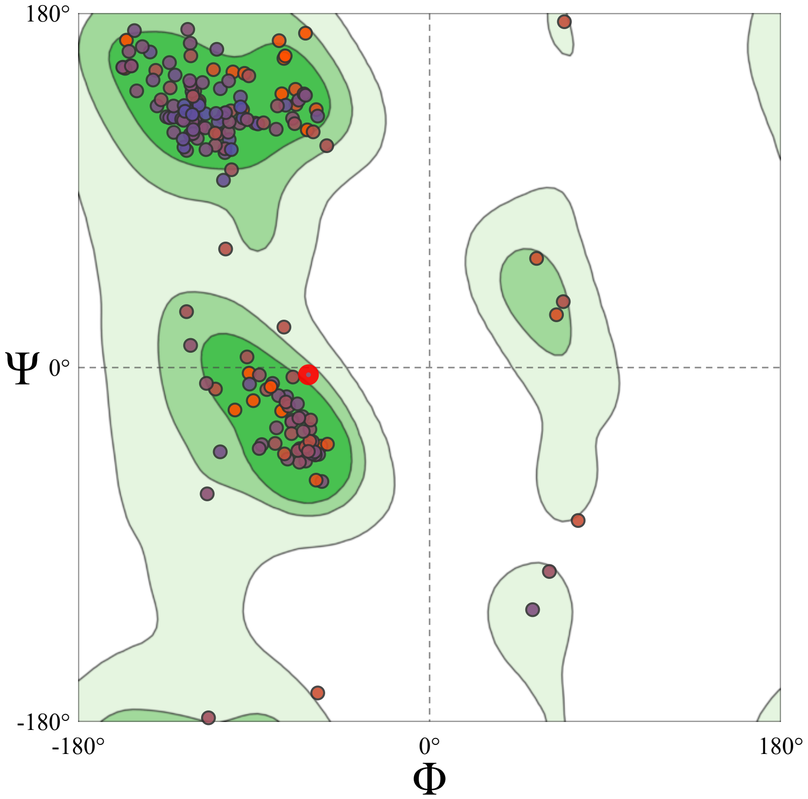

General Glycine Proline Pre-Proline Chain B

MolProbity Results

MolProbity is QUEUEING

Quality Estimate

QMEANDisCo Global: 0.64 ± 0.06

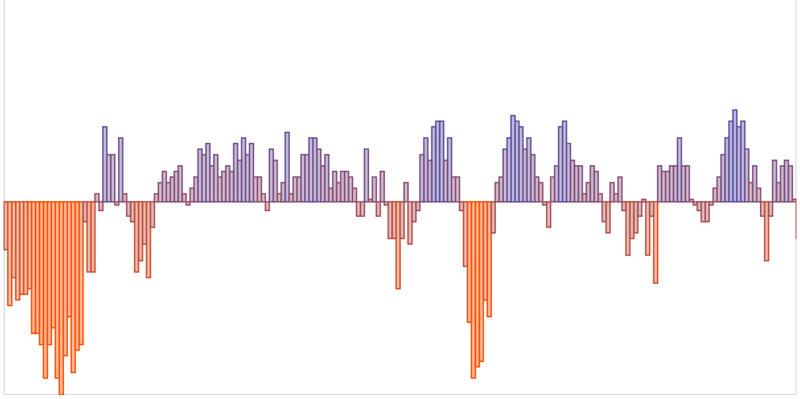

QMEAN Z-Scores

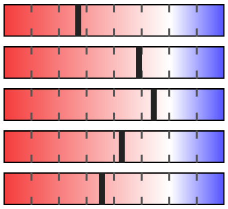

QMEAN	-3.30
Cβ	-1.10
All Atom	-0.56
solvation	-1.72
torsion	-2.44

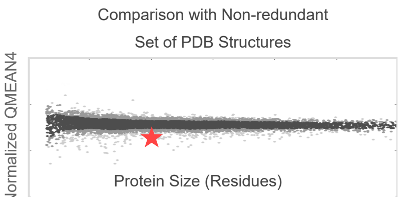

Residue Quality

```
     QMEAN
                                                21
  Chain:B MRIHSFFSLLFRLLLYSRADA---------
 4ah2.1.B MRMATPLLMQALPMGGGGSGGGGSGGGGSG   32

     QMEAN

  Chain:B -----LFGHGFIRCQFTSSNDTV-YLEQYY   45
 4ah2.1.B DTRPRFLWQLKFECHFFNGTERVRLLERCI   62

     QMEAN

  Chain:B INKMLLLQYNSTLGNVTGYTKKAIEIARDF   75
 4ah2.1.B YNQEESVRFDSDVGEYRAVTELGRPDAEYW   92
```

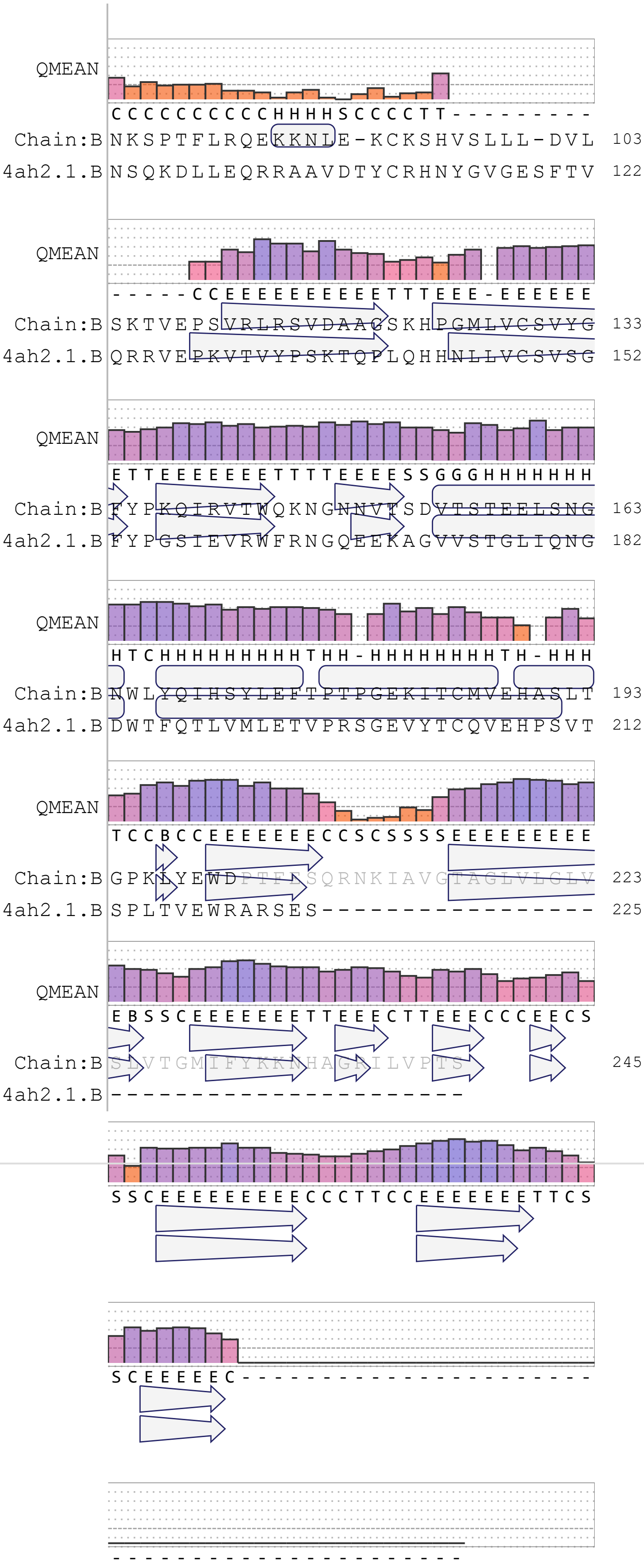
QMEAN
C C C C C C C C C C H H H H S C C C C T T - - - - - - - - -
Chain:B N K S P T F L R Q E K K N L E - K C K S H V S L L L - D V L 103
4ah2.1.B N S Q K D L L E Q R R A A V D T Y C R H N Y G V G E S F T V 122
QMEAN
- - - - - C C E E E E E E E E E E E T T T E E E - E E E E E E
Chain:B S K T V E P S V R L R S V D A A G S K H P G M L V C S V Y G 133
4ah2.1.B Q R R V E P K V T V Y P S K T Q P L Q H H N L L V C S V S G 152
QMEAN
E T T E E E E E E E T T T T E E E E S S G G G H H H H H H H
Chain:B F Y P K Q I R V T W Q K N G N N V T S D V T S T E E L S N G 163
4ah2.1.B F Y P G S I E V R W F R N G Q E E K A G V V S T G L I Q N G 182
QMEAN
H T C H H H H H H H H H T H H - H H H H H H H H T H - H H H
Chain:B N W L Y Q I H S Y L E F T P T P G E K I T C M V E H A S L T 193
4ah2.1.B D W T F Q T L V M L E T V P R S G E V Y T C Q V E H P S V T 212
QMEAN
T C C B C C E E E E E E E C C S C S S S S E E E E E E E E E
Chain:B G P K L Y E W D P T F L S Q R N K I A V G T A G L V L G L V 223
4ah2.1.B S P L T V E W R A R S E S - - - - - - - - - - - - - - - - - 225
QMEAN
E B S S C E E E E E E E T T E E E C T T E E E C C C E E C S
Chain:B S L V T G M I F Y K K N H A G R I L V P T S 245
4ah2.1.B -
S S C E E E E E E E E E E C C C T T C C E E E E E E E T T C S
S C E E E E E C -
- -

ASN 88 B

Confidence: 0.68

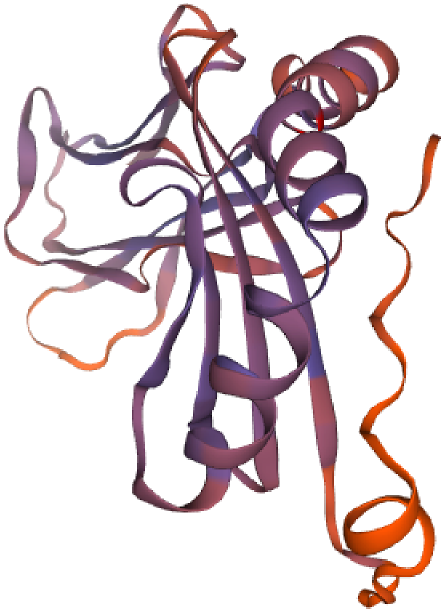

Cartoon
