# Supplementary material for: Comparative Reverse Vaccinology of Piscirickettsia salmonis, Aeromonas salmonicida, Yersinia ruckeri, Vibrio anguillarum and Moritella viscosa, Frequent Pathogens of Atlantic Salmon and Lumpfish Aquaculture
Source: Vaccines (Basel). 2022 Mar 18;10(3):473. doi: 10.3390/vaccines10030473 (PMC8954842; doi:10.3390/vaccines10030473)
Supplement: Supplementary file 1 [file vaccines-10-00473-s001.zip › Supplementary File S3_salmon_MHCII_refined.pdb _ Structure Assessment (1).pdf]

Structure Assessment

Help Examples

salmon_MHCII_refined.pdb;

Project Data

Created: Fri 14th Jan, 23:57;

We do not predict the uploaded structure to have a transmembrane segment.

Ramachandran Plots

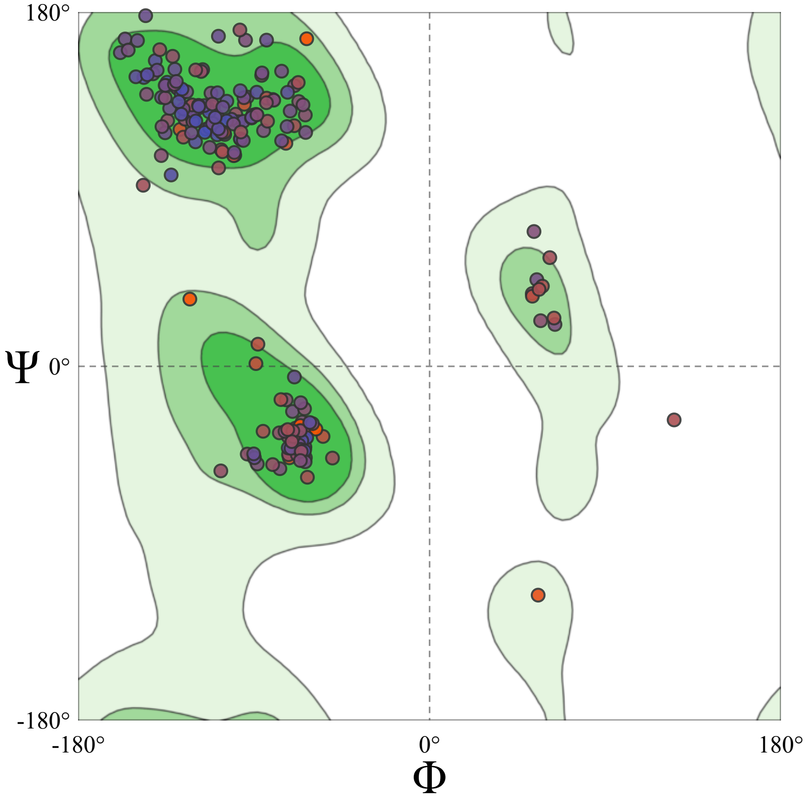

General Glycine Proline Pre-Proline All selected (2)

MolProbity Results

MolProbity Score	1.50	
Clash Score	9.35	(B23 PHE-B75 CYS)
Ramachandran Favoured	98.43%	

☐ Ramachandran Outliers	1.18%	A160 VAL, A73 TYR, A131 PRO
☐ Rotamer Outliers	0.92%	A131 PRO, B81 ILE
☐ C-Beta Deviations	9	A131 PRO, A160 VAL, A163 ASN, A122 LEU, A187 LEU, B44 TYR, A120 ASN, A155 TYR, A73 TYR
Bad Bonds	0 / 2097	
☐ Bad Angles	41 / 2857	B4 PHE, (A132 ALA-A133 PRO), B25 HIS, A131 PRO, A120 ASN, A122 LEU, (A159 ASP-A160 VAL), (A65 ASP-A66 PRO), (B79 ALA-B80 PRO), B5 TYR, A163 ASN, (A61 PRO-A62 PRO), B44 TYR, A77 VAL, B77 HIS, A47 TRP, A126 VAL, A187 LEU, A72 HIS, (A72 HIS-A73 TYR), A132 ALA, A155 TYR, A105 HIS, A22 HIS, A73 TYR, A125 HIS, B23 PHE, A63 PHE, (B2 GLY-B3 TYR), (A168 LEU-A169 PRO), A184 HIS, A164 GLN, A79 ASN, A156 PRO [illegible]
☐ Twisted Non-Proline	1 / 239	(A97 PRO-A98 GLU)

Results obtained using MolProbity version 4.4

Quality Estimate

QMEANDisCo Global: 0.68 ± 0.05

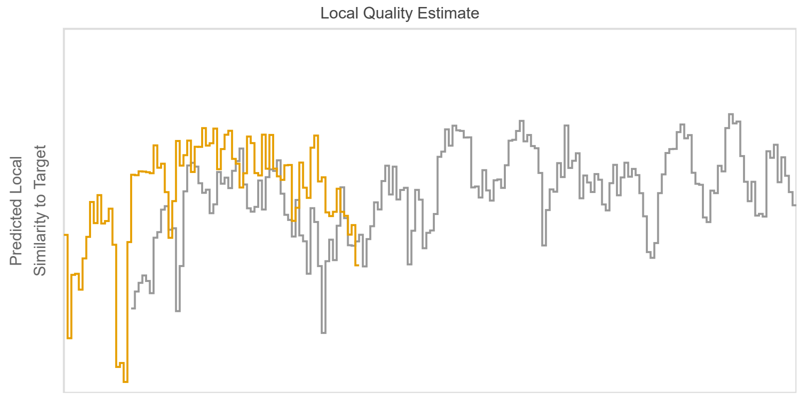

QMEAN Z-Scores

QMEAN	-1.47
Cβ	-0.61
All Atom	-0.55
solvation	-0.48
torsion	-1.27

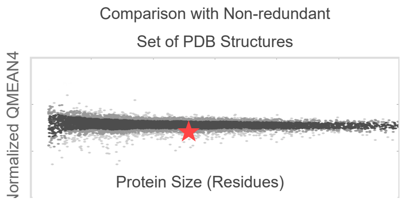

Residue Quality

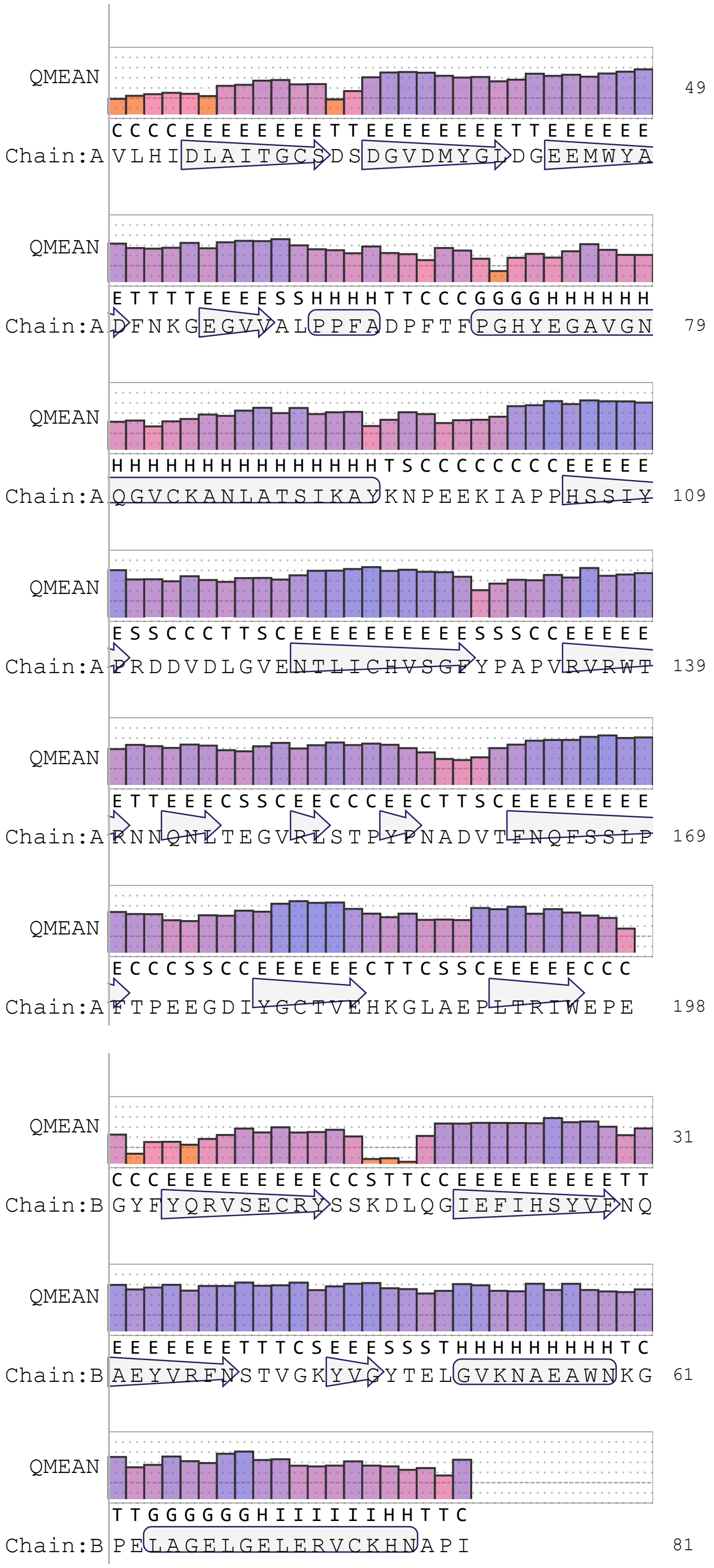

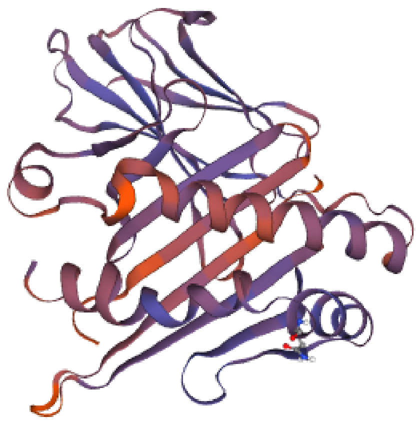

Cartoon
