# Supplementary material for: Comparative Reverse Vaccinology of Piscirickettsia salmonis, Aeromonas salmonicida, Yersinia ruckeri, Vibrio anguillarum and Moritella viscosa, Frequent Pathogens of Atlantic Salmon and Lumpfish Aquaculture
Source: Vaccines (Basel). 2022 Mar 18;10(3):473. doi: 10.3390/vaccines10030473 (PMC8954842; doi:10.3390/vaccines10030473)
Supplement: Supplementary file 1 [file vaccines-10-00473-s001.zip › Supplementary file S4_ramachandran_report_MHCII.pdf]

MolProbity Ramachandran analysis

model_4_1_trimmed.pdb, model 1

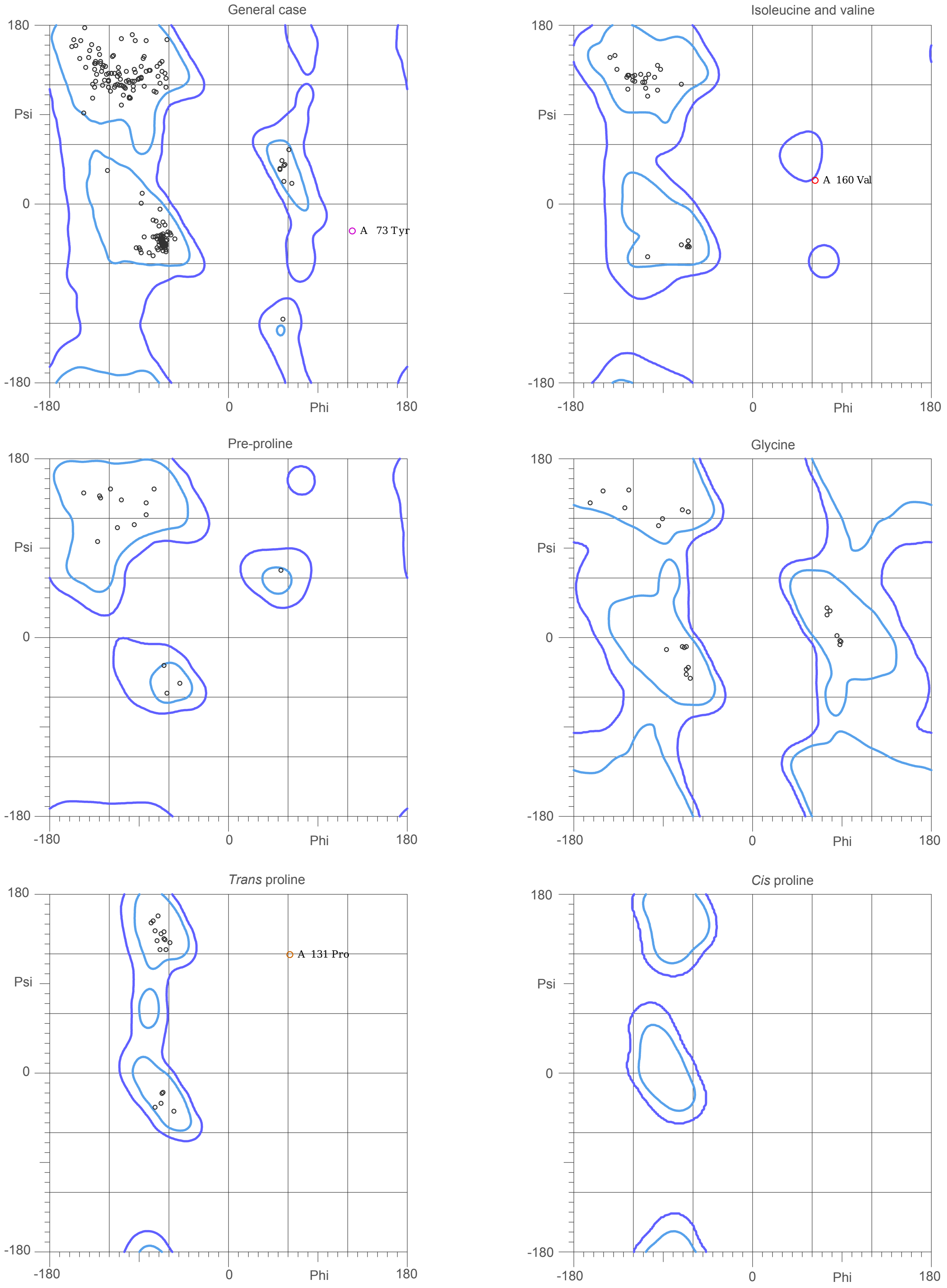

98.4% (251/255) of all residues were in favored (98%) regions.
98.8% (252/255) of all residues were in allowed (>99.8%) regions.

There were 3 outliers (phi, psi):
- A 73 Tyr (125.4, -27.3)
- A 131 Pro (62.1, 120.2)
- A 160 Val (63.8, 24.5)
