# Supplementary material for: Comparative Reverse Vaccinology of Piscirickettsia salmonis, Aeromonas salmonicida, Yersinia ruckeri, Vibrio anguillarum and Moritella viscosa, Frequent Pathogens of Atlantic Salmon and Lumpfish Aquaculture
Source: Vaccines (Basel). 2022 Mar 18;10(3):473. doi: 10.3390/vaccines10030473 (PMC8954842; doi:10.3390/vaccines10030473)
Supplement: Supplementary file 1 [file vaccines-10-00473-s001.zip › Supplementary tables1-5.pdf]

Table S1. Categories of MHC HLA-A (MHC class I) and HLA-DP and HLA-DQ (MHC class II) sequences present in the Vaxitop program used in this study.

MHCs	MHC Alleles
**MHC 1**	HLA-A*01:01, HLA-A*02:01, HLA-A*02:02, HLA-A*02:03, HLA-A*02:06, HLA-A*02:11, HLA-A*02:12, HLA-A*02:16, HLA-A*02:19, HLA-A*02:50, HLA-A*03:01, HLA-A*11:01, HLA-A*23:01, HLA-A*24:02, HLA-A*24:31, HLA-A*26:01, HLA-A*26:02, HLA-A*29:02, HLA-A*30:01, HLA-A*30:02, HLA-A*31:01, HLA-A*32:01, HLA-A*32:07, HLA-A*33:01, HLA-A*68:01, HLA-A*68:02, HLA-A*68:23, HLA-A*69:01, HLA-A*80:01, HLA-A11, HLA-A2, HLA-A3.
**MHC 2**	HLA-DPA1*01:03/DPB*02:01, HLA-DPA1*02:01/DPB*01:01, HLA-DPA1*02:01/DPB*05:01, HLA-DPA1*03:01/DPB*04:02, HLA- DPB*04:01, HLA-DPB*04:02, HLA-DQ2, HLA-DQA1*01:01/DQB1*05:01, HLA-DQA1*01:02/DQB1*06:02, HLA-DQA1*03:01/DQB1*03:02, HLA-DQA1*03:02/DQB1*04:01, HLA-DQA1*05:01/DQB1*02:01, HLA-DQA1*05:01/DQB1*03:01.

Table S2. Comparative reverse vaccinology outlining outer membrane antigens identified in study.

**Outer membrane antigens**	***P. salmonis***	***A. salmonicida***	***V. anguillarum***	***M. viscosa***	***Y. ruckeri***
OmpW family protein	+				
Porin family protein	+				
OmpA family protein	+			+	
DUF481 domain-containing protein	+				
Outer membrane protein assembly factor BamE	+	+			+
VacJ family lipoprotein	+				
phospholipase A	+				
LPS-assembly protein LptD	+	+	+	+	+
Outer membrane protein assembly factor BamA	+	+	+	+	+
Outer membrane protein transport protein	+	+	+	+	
TolC family outer membrane protein	+		+	+	
LbtU family siderophore porin	+				

TonB-dependent siderophore receptor	+	+	+	+	+
Autotransporter outer membrane beta-barrel domain-containing protein	+				+
Host specificity protein J	+				
Type IV conjugative transfer system lipoprotein traV		+			+
Maltoporin		+			+
Transporter		+			
Siderophore amonabactin TonB-dependent receptor		+			
TonB-dependent receptor		+	+	+	
Ligand-gated channel protein		+			
Porin		+	+	+	+
Glycine zipper 2TM domain-containing protein		+			
Peptidoglycan DD-metalloendopeptidase family protein		+			
Outer membrane protein assembly factor BamC		+			
DUF2860 family protein		+	+		
TonB-dependent hemoglobin/transferrin/lactoferrin family receptor		+	+	+	+
Membrane protein		+	+	+	
Flagellar basal body L-ring protein FlgH		+	+	+	+
Efflux transporter outer membrane subunit		+			
M23 family metallopeptidase		+	+		
Porin OmpAI		+			

Porin OmpAII	+			
Type IV pilus secretin PilQ family protein	+			
Type IV pilin biogenesis protein	+			
Carbohydrate porin	+	+		
Porin OmpA	+			
Conjugal transfer protein TraF		+	+	
MSHA biogenesis protein MshQ		+		
Murein hydrolase activator NlpD		+		+
Outer membrane beta-barrel protein		+	+	+
Luciferase		+		
Peptidase		+		
J domain-containing protein		+		
MipA/OmpV family protein		+		+
YjbH domain-containing protein		+		
TonB-dependent vitamin B12 receptor		+		
Sphingomyelin phosphodiesterase		+		
Retention module-containing protein		+		
DUF1566 domain-containing protein			+	
Outer membrane protein assembly factor BamD			+	
DUF1983 domain-containing protein			+	
DUF748 domain-containing protein			+	
DUF3466 family protein			+	
DUF1302 domain-containing protein			+	
TonB-dependent receptor plug domain-containing protein			+	
Maltoporin LamB			+	
Protein family protein			+	

LruC domain-containing protein	+	
Conjugal transfer protein TraN		+
Type-F conjugative transfer system mating-pair stabilization protein TraN		+
Outer membrane porin, OprD family		+
Outer membrane usher protein		+
Ion channel protein Tsx		+
Fimbrial biogenesis outer membrane usher protein		+
OmpA family lipoprotein		+
TonB-dependent copper receptor		+
Ig-like domain-containing protein		+
Hemagglutinin repeat-containing protein		+
Outer membrane channel protein TolC		+
Murein transglycosylase A		+
Envelope stress response activation lipoprotein NlpE		+
Rcs stress response system protein RcsF		+
Fimbria/pilus outer membrane usher protein		+
Llong-chain fatty acid transporter FadL		+
Porin OmpC		+
Ligand-gated channel protein		+
Lipid IV(A) palmitoyltransferase PagP		+
Outer membrane protein OmpW		+
Porin OmpF		+
Outer membrane protein OmpX		+

Table S3. Comparative reverse vaccinology outlining secreted antigens identified in this study.

**Secreted Antigens**	***P. salmonis***	***A. salmonicida***	***V. anguillarum***	***M. viscosa***	***Y. ruckeri***
Peptidase M4 family protein	+				
LbtU family siderophore porin	+				
DUF4402 domain-containing protein	+				
DUF1561 family protein	+				
C1 family peptidase	+				
Flagellar hook assembly protein FlgD	+	+	+	+	+
Flagellar hook-basal body complex protein	+				
Flagellar basal-body rod protein FlgG	+	+	+	+	+
Flagellar hook-associated protein FlgK	+	+			+
Flagellar hook-associated protein FlgL	+				+
Endonuclease/exonuclease/phosphatase family protein	+				
Cadherin-like domain-containing protein	+				
Calcineurin-like phosphoesterase	+				
DUF4280 domain-containing protein	+				
Flagellar filament capping protein FliD	+	+		+	+
B-type flagellin	+				
TAXI family TRAP transporter solute-binding subunit		+			
Flagellin		+	+	+	
DUF3466 family protein		+			
M6 family metalloprotease domain-containing protein		+			

Type I secretion C-terminal target domain-containing protein	+			
Phage tail protein	+			+
Lipase	+			
Beta-barrel pore-forming toxin aerolysin	+			
Type 1 fimbrial protein	+			+
Fimbrial protein	+			+
Peptidase M66	+			
Flagellar hook protein FlgE	+	+	+	
Flagellar hook-length control protein FliK	+		+	+
M20/M25/M40 family metallo-hydrolase	+			
Retention module-containing protein	+			
Type IV pilin protein	+			
N-acetylglucosamine-binding protein GbpA	+			+
Pilin	+	+		
Lateral flagellin LafA	+			
Lateral flagellar hook-associated protein LfgK	+			
Lateral flagellar basal-body rod protein LfgG	+			
Lateral flagellar hook protein FlgEL	+			
M4 family metallopeptidase		+	+	
Trypsin-like peptidase domain-containing protein		+		
Hcp family type VI secretion system effector		+		
SGNH/GDSL hydrolase family protein		+	+	
Immune inhibitor A		+		
Class C beta-lactamase		+		
Nucleotidyltransferase		+		

Hemolysin	+		
Endonuclease	+	+	
Deoxyribonuclease I	+		
Exo-alpha-sialidase	+		
Chitinase	+		
Ig-like domain-containing protein		+	
Triacylglycerol lipase		+	
Transferrin-binding protein-like solute binding protein		+	
Collagenase		+	
Membrane-targeted effector domain-containing toxin		+	
DUF1566 domain-containing protein		+	
Type II secretion system protein		+	
Lytic polysaccharide monooxygenase		+	+
Sphingomyelin phosphodiesterase		+	
Methyltransferase		+	
ABC transporter substrate-binding protein		+	+
Flagellar basal body protein FlgE		+	+
Aerolysin family beta-barrel pore-forming toxin		+	
Rcin-type beta-trefoil lectin domain protein		+	
DUF3570 domain-containing protein		+	
Conjugal transfer protein TraN			+
DUF481 domain-containing protein			+
Fimbria/pilus periplasmic chaperone			+
Heme acquisition hemophore HasA			+

Spore coat protein U domain-containing protein					+
Tail fiber protein					+
FliC/FljB family flagellin					+
Patatin-like phospholipase RssA					+
M10 family metallopeptidase					+
Peptidase M4 family protein	+				
LbtU family siderophore porin	+				
DUF4402 domain-containing protein	+				
DUF1561 family protein	+				
C1 family peptidase	+				
Flagellar hook assembly protein FlgD	+	+	+	+	+

Table S4. Selected B and T (MHC I and II) cell epitopes identified for the common antigens.

Antigens	Epitopes			aa
	**B CELL**	**MHC I**	**MHC II**	
TonB-dependent siderophore receptor	TANAT (14-18)	LMTKKTANA(9-17), NATKITRTF(16-24), KTANATKITR (13-23)	LMTKKTANA (9-17)	
	AKATSEPDQEPKHKKYNEKSATIPSSAKISSE (39-70)	TSEPDQEPK(42-50), ATIPSSAKI(59-67), KYNEKSATI (53-61)		39-70
	Y (171), Q (173)	YSQTGLSLF (171-179)		171-179
	PIPNQKTTT (215-223)	TLKQSVTAA (223-231)		215-233
	YWPSETTEM (283-291)	ETTEMKANL (287-295)		283-295
	DADAELPGALTPEQYNNNWRQSNTPDNRY (299-327)	NTPDNRYQA(321-329), RQSNTPDNRY (318-327)	PEQYNNNWR(310-318)	299-327
	TPT (343-345)	IISHDFTPT (337-345)		337-345
	FSWQDPGIAGVTPTDIAQS (361-379)	RNFSWQDPGI (359-368)	FSWQDPGIA(361-369)	359-379
	VY (419-420)	VYTDNIATV (419-427)		419-427
	AAY (443-445)	STNAYAAYA (438-446)		438-446
	QVANSNGQSLA (519-529)	QVANSNGQSL (519-528)		519-529

	H (586)	TQLHYTPDF (583-591)		583-591
	IQGENKGNQMPYA (609-621)	YAAKNQFSFI (620-629)		609-629
	FSDAANTTTEQTTQGPIP (650-667)	TTQGPIPNY (661-669)		650-669
	SQGITPAPGRS (710-720)	ITPAPGRSF (713-721)		710-721
LPS-assembly protein LptD	YSSTPTTKTKTPTKT (25-39)	GLLPSLTYS(18-26), SLTYSSTPT (22-30)	PTKTALDWV (36-44)	22-44
	H (50)	DWVASNKNHL (42-51)		42-51
	ALPGTPGPFNQS (71-82)	ALPGTPGPF (71-79)		71-82
	TYISS (84-88)	QSKTYISSK (81-89)		84-89
	STTLTPSGQTL (90-100)	TLTDNVYLY (99-107)		90-107
	TRDPKTGDMTQI (121-132)	TQINATGHVR (130-139)		121-139
	HWQPQKQQAQI (153-163)	KQQAQINDV (158-166)	PQKQQAQIN (156-164)	153-166
	DRSSQKVPANNTQLSNPGTGYAHGSATTVSQQ(175-206)	KVPANNTQL(180-188), YAHGSATTV (195-203)		175-206
	YSTCAPIPGQTW (218-229)	CAPIPGQTW (221-229)		218-229
	QTGRGEA (241-247)	NQQTGRGEAY (239-248)		239-248
	NNQRQ (270-274)	YFNFPINNQR (264-273)		264-274
	GLS (289-291)	SLTTPYYLNLA (291-301)		289-301
	AYGN (345-348)	KRYNVFFGQ (349-357)		345-357
	TQFT (359-362)	TQFTPNLNV (359-367)		359-367
	LA (490-491)	NTLANSANI (388-396)		388-491
	YQFPDPTLS (421-429)	TLSLGNRYYA (427-436)		421-436
	S (488), QTDF (490-493)	WSGQTDFGY (487-495)		487-495
	NNNTQGQDHL (510-519)	GQDHLSRGI (515-523)		510-523
	YKQT (545-548)	RYKQTLEPR (544-552)		544-552
	EQI (613-615)	ALINNNGEQI (606-615)		606-615
	THDNENY (696-702)	HIFNVGYTH (689-697)		689-702
	GVAPPNTKGL (713-722)	SQDELINGV(706-714), GLYSSMYWA (721-729)		706-729
outer membrane protein assembly factor BamA	WAA (26-28)	WAAPAGFVI (26-34)		26-34
	VK (56-57)	DLVLTNLPVK (48-57)		48-57

HHSI (110-113)	IVTEGHHSI (105-113)		105-113
NDKYTKPKLDT (219-229)		SFITDNDKY (214-222)	214-229
TPVPEVNKDNH (333-343)	EVNKDNHTV (337-345)		333-345
KATTPVVPGKPGYV (409-422)	KPGYVNVDYK (418-427)		409-427
PNVLG (458-462)	NVLGTGNTL (459-467)	GLGYSTPNV (452-460)	452-467
PYWTESG (487-493)	FTDPYWTES (484-492)		484-493
NKTNAEEQGLADYSTNSY (503-520)		EEQGLADYS (508-516), EEQGLADYS (508-516)	503-520
LNQGTNNSV (547-555)	NSVTVQNFI (553-561)		547-561
KWPFPTNGEKLS (586-597)	YTNLDKWPF (581-589)		581-597
GYGNSYGSGKGG (637-648)	GSGKGGLPF (643-651)	GLPFFNNFG (648-656)	637-656
AGGWSGSPTW (657-666)	PTWGMIRGY (664-672)		657-672
TLGPNDTIACSDGTQTCEGNA (674-694)	DTLGPNDTI (673-681)	TCEGNAIGG(689-697)	673-697
VYDTTTNRDTYNTANS (731-746)	VYDTTTNRD (731-739)		731-746
NYLGDKSPSL (749-758)	KSPSLSNLAY (754-763)		749-763

Table S5. Conserved putative domains of qualifying vaccine candidates.

Protein	Conserved Domain	Accession	Interval	E-Value
**LPS-assembly protein LptD**	LptD	COG1452	75-823	9.29E-107
	PRK04423	PRK04423	91-828	1.20E-94
	PRK03761	PRK03761	80-826	2.72E-77
	OstA_C	pfam04453	348-734	4.02E-74
	OstA	pfam03968	85-141	2.95E-04
**TonB-dependent siderophore receptor**	FecA	COG4772	85-729	7.72E-139
	TonB-siderophor	TIGR01783	99-729	1.04E-83
	ligand_gated_channel	cd01347	105-729	2.36E-70

CirA	COG1629	89-729	1.29E-45
TonB-hemin	TIGR01785	89-729	2.90E-41
FepA	COG4771	90-729	2.66E-36
TonB-hemlactrns	TIGR01786	95-729	1.92E-35
TonB_dep_Rec	pfam00593	280-728	1.72E-30
BtuB	COG4206	105-729	2.10E-29
TonB-B12	TIGR01779	105-729	4.22E-26
Plug	pfam07715	95-208	6.55E-25
PRK10064	PRK10064	90-700	7.07E-23
Fiu	COG4774	100-729	3.85E-22
PRK13483	PRK13483	90-729	7.63E-22
PRK13486	PRK13486	90-624	2.08E-21
PRK13528	PRK13528	63-729	1.41E-18
PRK13513	PRK13513	58-729	1.01E-17
PRK13484	PRK13484	50-646	9.26E-15
PRK13524	PRK13524	105-729	7.67E-14
FhuE	COG4773	100-729	1.74E-13
PRK09840	PRK09840	104-729	2.79E-11
btuB	PRK10641	105-214	5.09E-11
OMP_b-brl_3	pfam14905	440-726	1.75E-07
PRK10044	PRK10044	455-702	3.41E-05
TonB-Xanth-Caul	TIGR01782	421-729	6.35E-05
PRK14050	PRK14050	89-214	1.28E-04
PRK14049	PRK14049	64-214	6.69E-03

**Outer membrane protein assembly factor BamA**	OM_YaeT	TIGR03303	32-805	0.00E+00
	BamA	COG4775	9-805	0.00E+00
	PRK11067	PRK11067	9-805	1.61E-165
	Bac_surface_Ag	pfam01103	459-805	1.21E-58
	TamA	COG0729	320-805	1.30E-26
	POTRA	pfam07244	184-271	1.65E-13
	POTRA	pfam07244	274-354	1.99E-10
	POTRA	pfam07244	100-180	2.08E-09
	POTRA	pfam07244	357-430	1.65E-08
	POTRA	pfam07244	32-99	3.38E-04
**Flagellar hook assembly protein FlgD**	flgD	PRK06655	1-227	4.17E-74
	FlgD	COG1843	4-227	2.48E-51
	flgD	PRK12634	1-223	3.23E-43
	FLgD_tudor	pfam13861	90-223	8.92E-39
	flgD	PRK12633	20-226	3.10E-31
	flgD	PRK12813	34-226	2.44E-25
	FlgD	pfam03963	6-80	4.24E-23
	flgD	PRK09618	5-113	1.37E-20
	flgD	PRK12812	16-177	1.65E-19
	FlgD_ig	pfam13860	112-179	1.31E-16
	flgD	PRK09619	33-227	1.38E-14
	flgD	PRK06009	5-101	7.56E-13
	flgD	PRK11911	29-114	9.95E-11
	flgD	PRK05842	19-203	1.70E-09
	flgD	PRK06792	20-114	1.79E-08
	DUF2271	pfam10029	152-188	7.39E-05

**Flagellar basal-body rod protein**	flgG	PRK12693	1-259	########
**FlgG**	flgG_G_neg	TIGR02488	4-259	########
	flgG	PRK12691	4-259	########
	flgG	PRK12694	1-259	########
	FlgG	COG4786	1-259	1.59E-98
	flgG	PRK12816	1-254	1.39E-82
	flgG	PRK12692	4-261	1.11E-81
	FlgEFG_subfam	TIGR03506	4-243	2.49E-79
	flgG	PRK12636	18-257	2.21E-58
	flgG	PRK12817	1-258	8.83E-47
	flgE	PRK05682	4-255	6.47E-39
	flgF	PRK12690	19-242	3.55E-38
	flgF	PRK12640	1-259	9.51E-37
	flgF	PRK12689	11-253	3.62E-36
	FlgE	COG1749	1-257	1.65E-35
	FlgF	COG4787	1-261	7.25E-35
	flgG	PRK12818	1-255	3.01E-33
	Flg_bbr_C	pfam06429	182-259	2.20E-23
	flgE	PRK08425	1-133	5.95E-23
	flgE	PRK06803	7-261	3.60E-21
	flgF	PRK12642	1-255	1.24E-20
	flgE_epsilon	TIGR02489	1-133	7.90E-20
	flgF	PRK12641	18-261	5.01E-17
	flgE	PRK08425	170-259	8.81E-17
	flgE	PRK12637	147-257	2.55E-15
	flgF	TIGR02490	149-241	1.86E-14
	flgE_epsilon	TIGR02489	170-259	1.92E-13
	flgF	PRK12643	11-224	6.87E-13

flgG	PRK12819	5-251	1.49E-11
flgE	PRK05841	170-255	8.19E-11
flgC	PRK05681	204-255	5.81E-10
FlgC	COG1558	204-255	2.66E-09
flgE	PRK12637	10-133	1.21E-08
FlgC	TIGR01395	204-255	1.32E-08
flgE	PRK05841	8-133	5.17E-06
flgC	PRK05681	7-62	3.05E-05
FlgC	COG1558	7-43	3.87E-05
Flg_bb_rod	pfam00460	5-35	4.45E-05
flgK_ends	TIGR02492	4-83	1.89E-04
flgC	PRK12632	4-45	2.77E-04
FlgC	TIGR01395	7-61	2.95E-04
flgB	PRK12623	9-42	7.55E-04
flgC	PRK12782	4-33	1.37E-03
FlgK	COG1256	176-257	1.81E-03
flgK	PRK07739	5-43	2.96E-03
flgC	PRK12631	205-255	3.07E-03
flgK	PRK08471	211-257	5.48E-03
flgC	PRK12628	205-255	5.94E-03
flgC	PRK12630	7-42	8.58E-03
